# Trichostatin A Suppresses EGFR Expression through Induction of MicroRNA-7 in an HDAC-Independent Manner in Lapatinib-Treated Cells

**DOI:** 10.1155/2014/168949

**Published:** 2014-02-23

**Authors:** Chih-Yen Tu, Chia-Hung Chen, Te-Chun Hsia, Min-Hsiang Hsu, Ya-Ling Wei, Meng-Chieh Yu, Wen-Shu Chen, Ke-Wei Hsu, Ming-Hsin Yeh, Liang-Chih Liu, Yun-Ju Chen, Wei-Chien Huang

**Affiliations:** ^1^Division of Pulmonary and Critical Care Medicine, China Medical University and Hospital, Taichung 404, Taiwan; ^2^Department of Internal Medicine, China Medical University and Hospital, Taichung 404, Taiwan; ^3^School of Medicine, China Medical University, Taichung 404, Taiwan; ^4^Department of Life Science, National Chung-Hsing University, Taichung 402, Taiwan; ^5^Department of Respiratory Therapy, China Medical University, Taichung 404, Taiwan; ^6^Graduate Institute of Clinical Medical Science, China Medical University, Taichung 404, Taiwan; ^7^Graduate Institute of Cancer Biology, China Medical University, Taichung 404, Taiwan; ^8^Center for Molecular Medicine, China Medical University and Hospital, Taichung 404, Taiwan; ^9^Department of Pharmacology, College of Medicine, National Taiwan University, Taipei 106, Taiwan; ^10^Department of Biological Science and Technology, China Medical University, Taichung 404, Taiwan; ^11^Section of Breast Surgery, China Medical University and Hospital, Taichung 404, Taiwan; ^12^Department of Surgery, China Medical University and Hospital, Taichung 404, Taiwan; ^13^Department of Medical Research, E-Da Hospital, Kaohsiung 824, Taiwan; ^14^Department of Biological Science & Technology, I-Shou University, Kaohsiung 824, Taiwan; ^15^The Ph.D. Program for Cancer Biology and Drug Discovery, China Medical University, Taichung 404, Taiwan; ^16^Department of Biotechnology, Asia University, Taichung 413, Taiwan

## Abstract

Lapatinib, a dual EGFR/HER2 tyrosine kinase inhibitor, has been shown to improve the survival rate of patients with advanced HER2-positive breast cancers. However, the off-target activity of lapatinib in inducing EGFR expression without tyrosine kinase activity was demonstrated to render HER2-negative breast cancer cells more metastatic, suggesting a limitation to the therapeutic effectiveness of this dual inhibitor in HER2-heterogeneous tumors. Therefore, targeting EGFR expression may be a feasible approach to improve the anticancer efficiency of lapatinib-based therapy. Inhibition of HDAC has been previously reported to epigenetically suppress EGFR protein expression. In this study, however, our data indicated that treatment with HDAC inhibitors trichostatin A (TSA), but not suberoylanilide hydroxamic acid (SAHA) or HDAC siRNA, can attenuate both protein and mRNA expressions of EGFR in lapatinib-treated triple-negative breast cancer cells, suggesting that TSA may suppress EGFR expression independently of HDAC inhibition. Nevertheless, TSA reduced EGFR 3′UTR activity and induced the gene expression of microRNA-7, a known EGFR-targeting microRNA. Furthermore, treatment with microRNA-7 inhibitor attenuated TSA-mediated EGFR suppression. These results suggest that TSA induced microRNA-7 expression to downregulate EGFR expression in an HDAC-independent manner.

## 1. Introduction

Amplification and overexpression of HER2 (also named ErbB2) receptor tyrosine kinase, detected in 20–30% of breast cancer, are associated with a poor clinical patient outcome, including lymph node metastasis, shorter survival, and shorter time to recurrence [1, 2]. Activation of HER2 initiates a cascade of signal transduction, including PI3K/Akt and MAPK pathways, to mediate cell growth and survival [3]. The dysregulation of these signal pathways from the overexpressed HER2 elicits multiple gene transcriptions associated with neoplastic transformation, initiation, cellular immortalization, and tumor progression [4]. Thus, targeting the tyrosine kinase activity of this receptor is viewed as promising therapeutic strategy to treat breast cancer patients with HER2 overexpression [3, 5].

Lapatinib (Tykerb, GW-572016), a dual tyrosine kinase inhibitor of epidermal growth factor receptor (EGFR) and HER2 receptors, has been used for advanced HER2-positive breast cancer patients who failed to chemotherapy or HER2-targeted therapy with monoclonal antibody trastuzumab [6, 7]. Although the majority of clinical benefits from lapatinib-based treatment were observed in patients with HER2-positive breast cancers, there are still several clinical trials of lapatinib in HER2-negative patients due to its EGFR inhibition activity [8–16]. Expression of EGFR has been found in up to 80% of triple-negative (HER2/ER/PgR-negative) breast cancers, and targeting EGFR thus has also been viewed as a potential therapeutic strategy for such disease [17–20]. When used as a monotherapy or in combination with chemotherapies, the clinical benefits of lapatinib in triple-negative or HER2-negative breast cancers have been tested in phase II trials [21, 22]. However, no significant benefit derived from the addition of lapatinib to paclitaxel was found in overall HER2-negative diseases, and surprisingly a worse clinical outcome with shorter median even-free survival was even found in breast cancer patients with triple-negative or HER2-negative/PgR-negative tumors [14]. Our previous study further uncovered an off-target activity of lapatinib in promoting the aggressiveness of triple-negative cell lines to axillary lymph node and lung in orthotopic tumor-xenograft mice [23]. Elevation of EGFR through downregulation of microRNA-7 [24] has been demonstrated to contribute to the lapatinib-increased cell motility. Therefore, targeting EGFR protein expression would be an effective strategy to prevent the lapatinib-elicited cell metastasis.

Histone deacetylases (HDACs), which regulate gene transcriptions by removing the acetyl groups from lysine residues of histones or transcription factor proteins, were frequently overexpressed in a variety of cancer types [25]. Higher expression of several HDAC subtypes was associated with enhanced migration and invasion of breast cancer cells [26–28]. The prometastatic effects of HDACs are connected to the transcriptional regulation of EGFR [29]. By suppressing EGFR expression, HDAC inhibitors were also shown to possess antitumor [30] and antidiabetes-associated kidney growth [31] activities and to synergize the anticancer activity of EGFR tyrosine kinase inhibitor gefitinib [29]. But the molecular mechanisms of HDAC inhibitor-reduced EGFR expression remain largely unknown. Thus, these open questions prompted us to investigate whether and how HDAC inhibitors suppress the lapatinib-induced EGFR expression.

In this study, we unexpectedly found that HDAC inhibitor trichostatin A (TSA), but not suberoylanilide hydroxamic acid (SAHA), represses EGFR protein level independently of HDAC inhibition in the lapatinib-treated breast cancer cells. Regardless of its HDAC inhibition activity, TSA induced microRNA-7 to target EGFR 3′UTR. These results discovered an off-target activity of TSA in regulating microRNA expression.

## 2. Materials and Methods 

### 2.1. Cell Lines, Constructs, Antibodies, and Reagents

Human breast cancer cell lines MDA-MB-231 and their derivatives were cultured in DMEM/F-12 with 10% fetal bovine serum. Lapatinib-selected cancer cells were established by selection with gradually increasing concentrations of lapatinib for over two months. Established resistant cancer cell lines were tested for their insensitivity to the corresponding drug and were cultured in the presence of 1 *μ*M lapatinib. HDAC siRNA clones were purchased from Dharmacon. Cells were transfected with siRNA oligo (5′-GAUGCUGAACCAUGCACCUTT-3′) and (5′-CACCAUGCAGAUCAUUCAATT-3′) to target HDAC3 and HDAC7, respectively, or with nontargeting control siRNA (5′-UGGUUUACAUGUCGACUAA-3′) with DharmaFECT 1 (Dharmacon) for 72 hrs for further experiments. Anti-EGFR (SC-03), anti-HDAC3, anti-HDAC7, and antiactin antibodies from Santa Cruz were used for Western blot analysis. HDAC inhibitors (TSA and SAHA), proteasomal inhibitors, and lysosomal inhibitors were purchased from Sigma-Aldrich (St. Louis, MO, USA).

### 2.2. Western Blot Analysis

Total cell lysates were prepared and subjected to SDS-PAGE using 7.5% running gels. The proteins were transferred to a polyvinylidene difluoride (PVDF) membrane, which was then incubated at room temperature for 1 h with 0.1% milk in TTBS (50 mM Tris-HCl, pH 7.5, 0.15 M NaCl, and 0.05% Tween-20), for 1 h with specific primary antibodies and for 30 min with HRP-labeled anti-rabbit antibody. After each incubation, the membrane was washed extensively with TTBS. The immunoreactive bands are detected using ECL detection reagent and Hyperfilm ECL (Amersham International).

### 2.3. RNA Isolation, Reverse Transcription (RT), and Real-Time Polymerase Chain Reaction (PCR)

Total RNA was extracted using TRIzol reagent (Invitrogen) according to the manufacturer's instructions as described previously [32]. For microRNA, each RT reaction contained 2 *μ*g of RNA, 50 nmol/L of the stem-loop RT primer, 0.25 mmol/L of each deoxynucleotide triphosphate, 50 units of Moloney murine leukemia virus reverse transcriptase (Invitrogen), 1 × RT buffer, 10 mmol/L DTT, and 4 units of RNase inhibitor. The stem-loop RT primer for hsa-miR-7 was designed according to mature miRNA sequence (Sanger Center miRNA Registry, http://www.mirbase.org/). The sequences of the RT primers are as follows: hsa-miR-7 RT primer, 5′-GTTGGCTCTGGTGCAGGGTCCGAGGTATTCGCACCAGAGCCAACACAACA-3′; U48 RT primer, 5′-GTTGGCTCTGGTGCAGGGTCCGAGGTATTCGCACCAGAGCCAACTCAGCG-3′. Real-time PCR reaction contained 0.5 *μ*mol/L of each forward and reverse primer, 0.1 *μ*mol/L of the Universal ProbeLibrary Probe #21 (Roche), the 1 × LightCycler TaqMan Master, and 2 *μ*L of cDNA using a Roche LightCycler 480 Real-Time PCR system. U48 small nuclear RNA was used as an internal control. The sequences of the forward primers were as follows: hsa-miR-7, 5′-GCGGCGTGGAAGACTAGTGAT-3′; U48, 5′-CGGCGGTAACTCTGAGTGTGT-3′. The reverse primer for all of the above sets of genes was 5′-GTGCAGGGTCCGAGGT-3′. For *EGFR* and *Actin* mRNA, 1 *μ*g of total RNA was subjected to RT with an oligo-dT primer using a reverse transcriptase kit (Invitrogen). Equal amounts of cDNA (2 *μ*L) were subjected to PCR and amplified with 30 cycles using the following primers: *EGFR*, forward 5′-GTTGATATCATGCGACCCTCCGGGACG-3′ and reverse 5′-GGTTCTAGATCATGCTCCAATAAATTC-3′; HDAC3, forward 5′-ATGAAGTCGGGGCAGAGAGTG-3′ and reverse 5′-CACAATGCACGTGGGTTGG-3′; HDAC7, forward 5′-TCTGTCCCGGGCTCAGTCTT-3′; *Actin*, forward 5′-CTGGAACGGTGAAGGTGACA-3′ and reverse 5′-AAGGGACTTCCTGTAACAATGCA-3′. The PCR products were subjected to 1.2% agarose gel electrophoresis and visualized by ethidium bromide staining. Real-time PCR reactions containing 0.3 *μ*L of cDNA, 0.3 *μ*L of the forward and reverse primers, 5 *μ*L of 2X SYBR Green (Roche), and 1.4 *μ*L of distilled water were performed with a Roche LightCycler 480 Real-Time PCR system.

### 2.4. Histone Deacetylase Activity Assay

The assay was performed according to the manufactures instruction (Enzo Life Sciences, Farmingdale, NY, USA). Nuclear extracts were prepared and subjected to immunoprecipitation with specific HDAC antibodies. A reaction of substrate deacetylation was initiated by mixing fluorescence-labeled acetylated peptide with HDAC-containing nuclear extract or immunoprecipitates within a set time period. The developer was then added to the reaction to cleave the resultant deacetylated fluorescence-labeled peptide and to stop HDAC activity, resulting in the production of the chemiluminescent compound. The enhancer was then added to make the visualization of the chemiluminescent product.

### 2.5. Transfection and Reporter Gene Assay

The luciferase reporter gene containing full-length 3′ untranslated region (UTR) of human *EGFR* gene was a gift from Dr. Keith Giles (Western Australian Institute for Medical Research). Cells with 60–80% of confluence were transfected with 0.5 *μ*g of EGFR-3′UTR luciferase plasmid by using TransIT-2020 transfection reagent according to the manufacturer's instruction. After 24 hrs of transfection, cells were treated with TSA for another 24 hrs and total lysates were harvested and subjected to luciferase activity assays. Luciferase activity was normalized to *β*-gal. For siRNA/microRNA transfection, cells with 60–80% of confluence were transfected with various siRNA/microRNA by using DharmaFECT 1 transfection reagent. Cells were harvested at indicated time points and subjected to further experiment.

### 2.6. Statistical Analysis

In vitro experiments are repeated thrice and statistical analysis is done using Student's *t*-test. Data are presented as mean ± SE. A probability level of a *P* value of <0.05 is considered significant.

## 3. Results

### 3.1. TSA Suppressed EGFR Expression Independently of HDAC Inhibition

**Figure 1:**
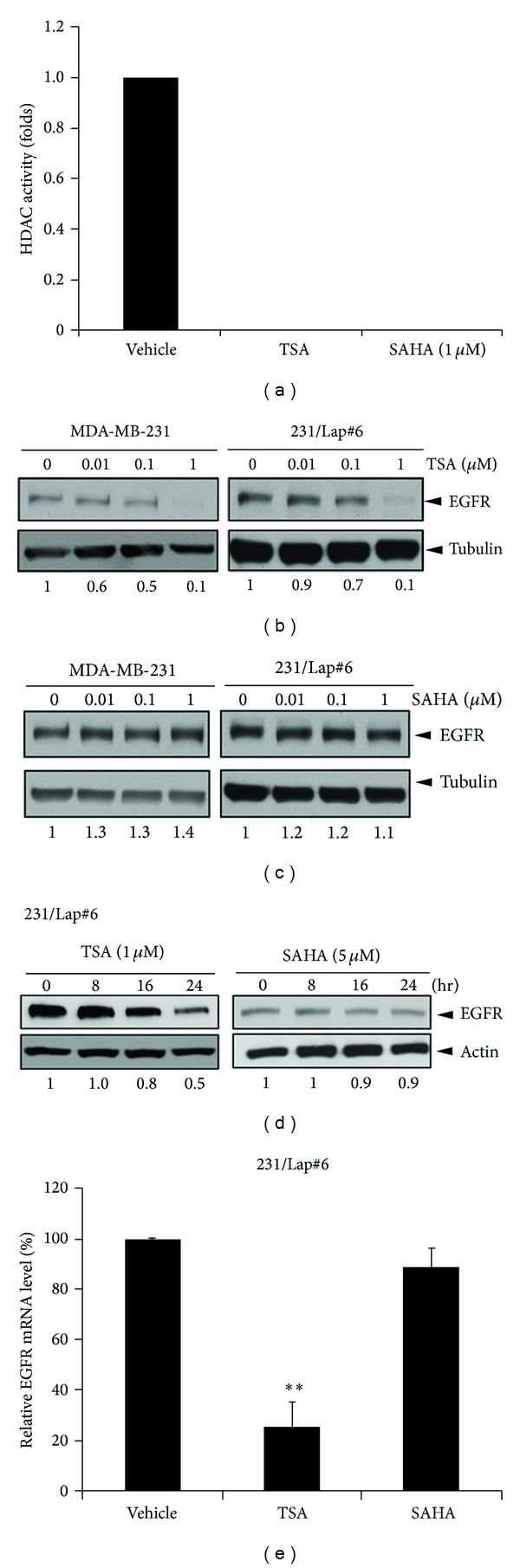
TSA but not SAHA suppressed lapatinib-induced EGFR expression. (a) Nuclear extract of HeLa cells was added with 1 mM TSA or SAHA for 30 min and then subjected to HDAC activity assays. (b) and (c) MDA-MB-231 and 231/Lap#6 cells were treated with indicated concentration of TSA (b) or SAHA (c) for 24 hours. Total lysates were prepared and subjected to Western blot analysis with indicated antibodies. (d) 231/Lap#6 cells were treated with 1 *μ*M TSA or 5 *μ*M SAHA for 8, 16, or 24 hours. Total lysates extracted from these cells were subjected to Western blot analysis with anti-EGFR and anti-actin antibodies. (e) 231/Lap#6 cells were treated with 1 *μ*M TSA or 5 *μ*M SAHA for 24 hours. Total RNA extracted from these cells was subjected to RT-qPCR with EGFR-specific primers. The induction of EGFR mRNA was normalized to GAPDH expression.

Our previous study showed that chronic treatment of triple-negative MDA-MB-231 breast cancer cells with lapatinib dramatically increased EGFR expression, which contributed to cell migration and invasion [23]. It led us to further study whether HDAC inhibitors possess the suppressive effect on EGFR expression in the lapatinib-treated MDA-MB-231 (231/Lap#6) cells. The IC50 of TSA or SAHA for HDAC inhibition is 0.01-0.02 *μ*M [33, 34], and treatments with TSA and SAHA at 1 *μ*M can completely abolish the total HDAC activity (Figure 1(a)). Treatment of both parental and 231/Lap#6 cells with pan-HDAC inhibitor TSA up to 1 *μ*M for 24 hours dramatically inhibited EGFR expression in a dose-dependent manner (Figure 1(b)). However, treatment with another pan-HDAC inhibitor SAHA up to 1 *μ*M did not affect the EGFR expression (Figure 1(c)). Moreover, treatment with TSA but not SAHA also time-dependently suppressed EGFR expression in 231/Lap#6 cells (Figure 1(d)). In parallel to the protein level, the mRNA level of EGFR in 231/Lap#6 cells was also reduced by treatment with TSA but not SAHA for 24 hours (Figure 1(e)). Since TSA and SAHA at 1 *μ*M showed the different effects on EGFR expression even though both of them can completely suppress the total HDAC activity at the same concentration, we next addressed whether inhibition of HDAC is involved in the suppression of EGFR by TSA in the lapatinib-treated cells.

**Figure 2:**
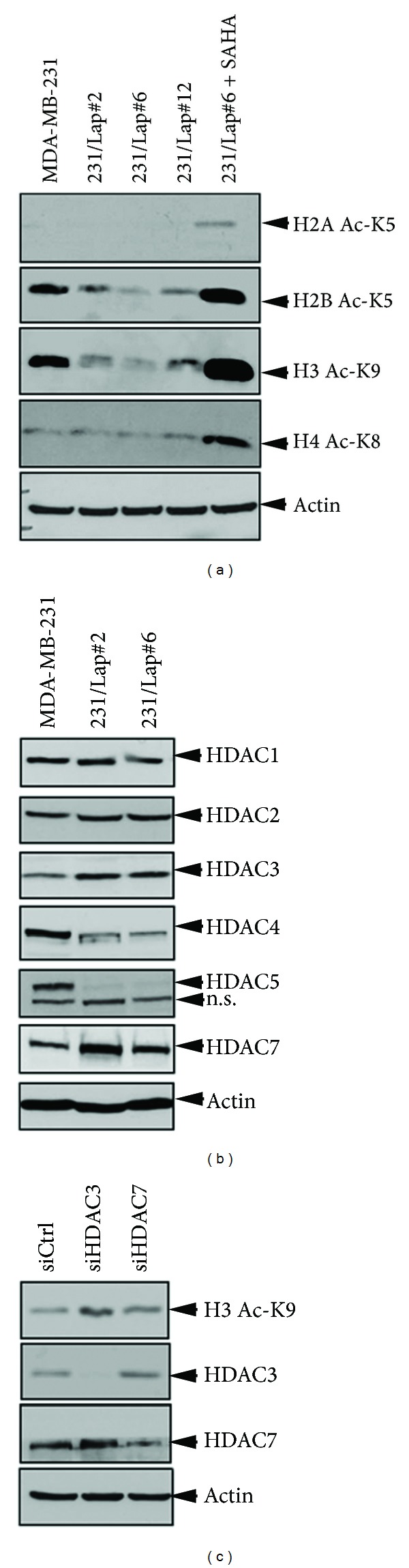
HDAC3 and HDAC7 expressions were elevated to cause histone hypoacetylations in lapatinib-treated MDA-MB-231 cell. (a) and (b) Total lysates from parental and various lapatinib-treated MDA-MB-231 cells were subjected to Western blot analysis with indicated antibodies. (c) Total lysates from 231/Lap#6 cells transfected with specific HDAC3 or HDAC7 siRNA for 72 hours were subjected to Western blot analysis.

**Figure 3:**
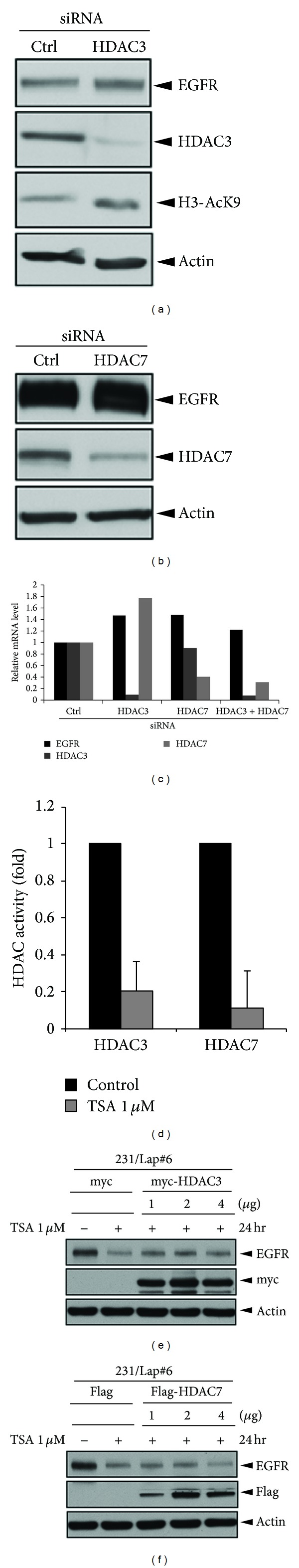
Silence of HDACs did not affect EGFR expression in lapatinib-treated MDA-MB-231 cells. (a)–(c) 231/Lap#6 cells were transfected with control siRNA or specific siRNA against HDAC3 ((a) and (c)) or HDAC7 ((b) and (c)) for 3 days. Total protein lysates prepared from these cells were subjected to Western blot analysis with indicated antibodies ((a) and (c)). Total RNA extracted from these cells was subjected to RT-qPCR. The relative mRNA levels of EGFR, HDAC3, and HDAC7 were normalized to GAPDH expression. (d) Total lysates of 231/Lap# cells transfected with myc-HDAC3 or myc-HDAC7 were subjected to immunoprecipitation with anti-myc antibody. The HDAC activities in the immunoprecipitates were measured in the HDAC activity assays. (e) and (f) 231/Lap#6 cells were transfected with increasing doses of myc-HDAC3 or myc-HDAC7 followed by treatment with TSA. Total lysates were prepared and subjected to Western blot.

In response to lapatinib treatment, the acetylations of histone H2B at K5 and H3 at K9 were suppressed in various lapatinib-treated clones of MDA-MB-231 cells (Figure 2(a)). In parallel to the downregulation of histone acetylation, our data also showed that the protein levels of HDAC3 and HDAC7 but not HDAC1, HDAC2, HDAC4, and HDAC5 were significantly increased in 231/Lap#6 cells (Figure 2(b)). Furthermore, silence of both HDAC3 and HDAC7 by siRNA can significantly restore the acetylation of histone H3 K9 (Figure 2(c)). These results suggest that the elevated HDAC3 and HDAC7 play a major role in lapatinib-mediated histone hypoacetylation at these residues. Therefore, we examine the regulation of EGFR expression by HDAC with focus on the elevated HDAC3 and HDAC7. However, silence of HDAC3 or HDAC7 did not affect the protein (Figures 3(a) and 3(b)) and mRNA (Figure 3(c)) levels of EGFR in 231/Lap#6 cells. Similar to SAHA, however, siRNA-mediated silence of HDAC3, which has been reported to contribute to EGFR transcription, did not change EGFR protein (Figure 3(a)) and mRNA (Figure 3(c)) expressions in 231/Lap#6 cells. Silence of HDAC7, a member of class IIa HDAC, also did not affect the protein and mRNA levels of EGFR (Figures 3(b) and 3(c)). To further confirm that these HDAC isoforms were not involved in the TSA-mediated EGFR suppression, HDAC3 and HDAC7 were ectopically overexpressed followed by treatment with TSA. Treatment of 231/Lap#6 cells with 1 *μ*M TSA suppressed the histone deacetylase activities of HDAC3 and HDAC7 (Figure 3(d)), but overexpression of myc-HDAC3 (Figure 3(e)) or myc-HDAC7 (Figure 3(f)) still did not restore the TSA-mediated EGFR suppression in 231/Lap#6 cells. These results suggest that TSA suppressed EGFR expression through an HDAC3/7-independent manner in 231/Lap#6 cells regardless of the changes in HDAC protein expression and histone acetylation in response to lapatinib.

### 3.2. TSA-Induced EGFR Suppression Did Not Involve Proteasomal and Lysosomal Degradation

**Figure 4:**
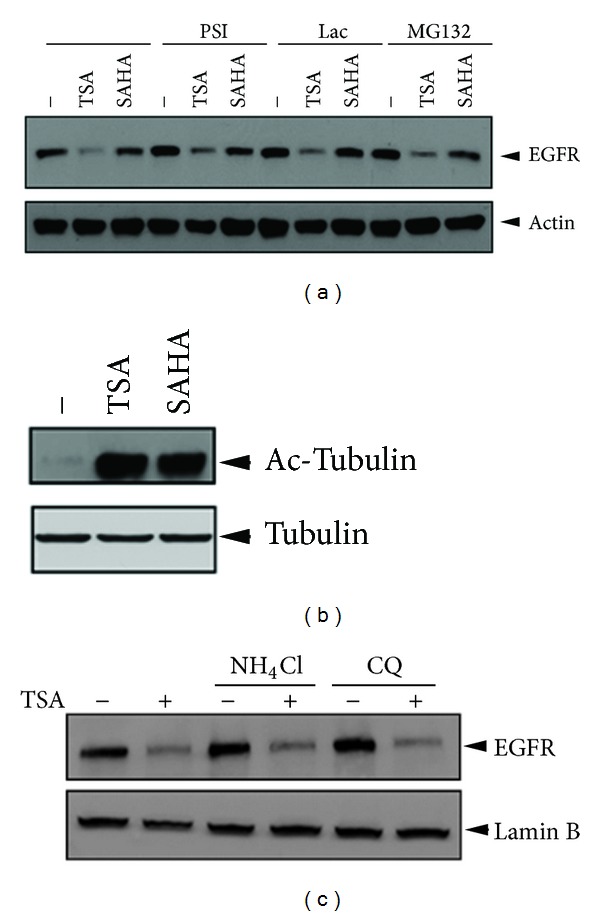
Proteasomal or lysosomal protein degradations were not involved in TSA-induced EGFR downregulation. 231/Lap#6 cells were pretreated with proteasomal inhibitor 5 *μ*M PSI, 5 *μ*M lac, and 1 *μ*M MG132 for 2 hrs (a) or with lysosomal inhibitor 1 *μ*M NH_4_Cl and 25 *μ*M CQ for 2 hrs (b) followed by treatment with 1 *μ*M TSA or 5 *μ*M SAHA for 24 hours. Total lysates were then extracted and subjected to Western blot analysis with indicated antibodies.

Treatment with HDAC inhibitors has been shown to induce ubiquitination of proteasomal degradation of erbB family to potentiate the antitumor activity of EGFR tyrosine kinase inhibitor in head and neck squamous tumors [29]. To test whether TSA suppressed EGFR expression in a proteasomal pathway, 231/Lap#6 cells were pretreated with proteasome inhibitors MG132, PSI, and lactacystin. However, the protein level of EGFR remains suppressed by TSA in the presence of these proteasomal inhibitors (Figure 4(a)), indicating that proteasomal degradation was not involved in the TSA-induced EGFR turnover. HDAC6, a cytoplasmic lysine deacetylase, was found to negatively regulate EGFR endocytosis and degradation by controlling the acetylation status of *α*-tubulin and subsequent EGFR trafficking along microtubules [35]. Therefore, loss of the microtubule-associated HDAC6 activity resulted in the EGFR lysosomal degradation through accelerating EGFR segregation from early endosomes to late endosomal and lysosomal compartments [35]. Next, we examined whether HDAC6 inhibition and proteasomal degradation are involved in TSA-mediated EGFR suppression. Both treatments with TSA or SAHA induced tubulin acetylation (Figure 4(b)), supporting their inhibitory effect on HDAC6. However, pretreatment with two lysosomal inhibitors, NH_4_Cl and chloroquine (CQ), still cannot prevent the TSA-mediated EGFR downregulation (Figure 4(c)), ruling out the possibility that TSA decreases EGFR expression through HDAC6-dependent lysosomal degradation.

### 3.3. TSA Attenuated EGFR Expression through Induction of miR-7 Expression

**Figure 5:**
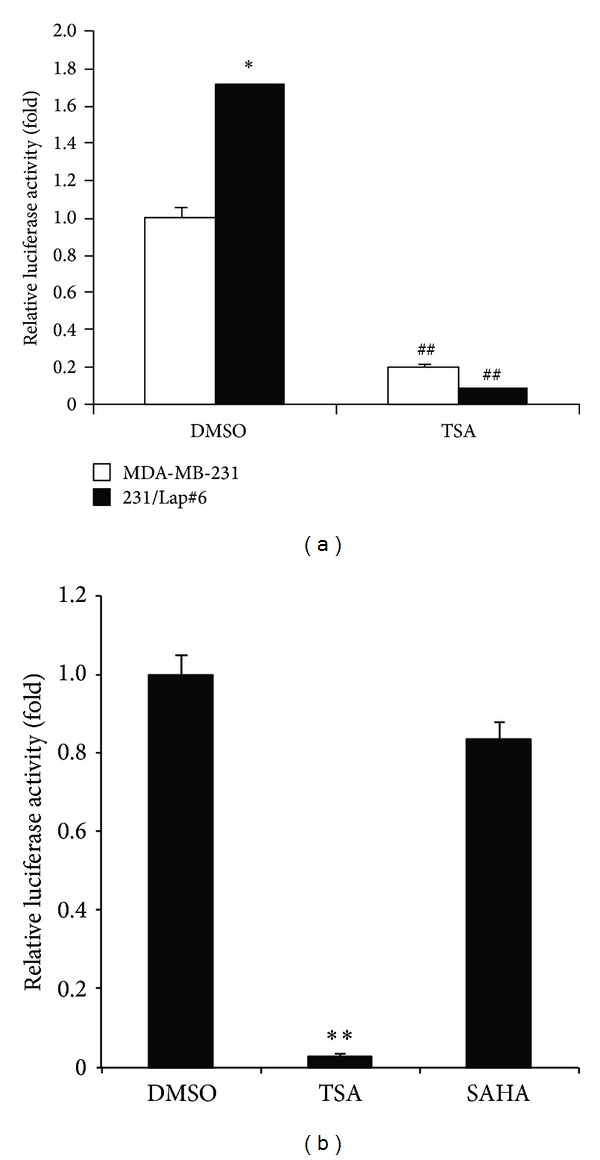
TSA suppressed the 3′UTR activity of *EGFR mRNA*. MDA-MB-231 and 231/Lap#6 cells were cotransfected with EGFR 3′UTR-luciferase plasmid and *β*-galactosidase for 24 hrs followed by treatment with 1 *μ*M TSA or SAHA for another 24 hrs. Total lysates were then prepared and subjected to luciferase and *β*-galactosidase activity assays. The relative luciferase activity was normalized to *β*-galactosidase.

**Figure 6:**
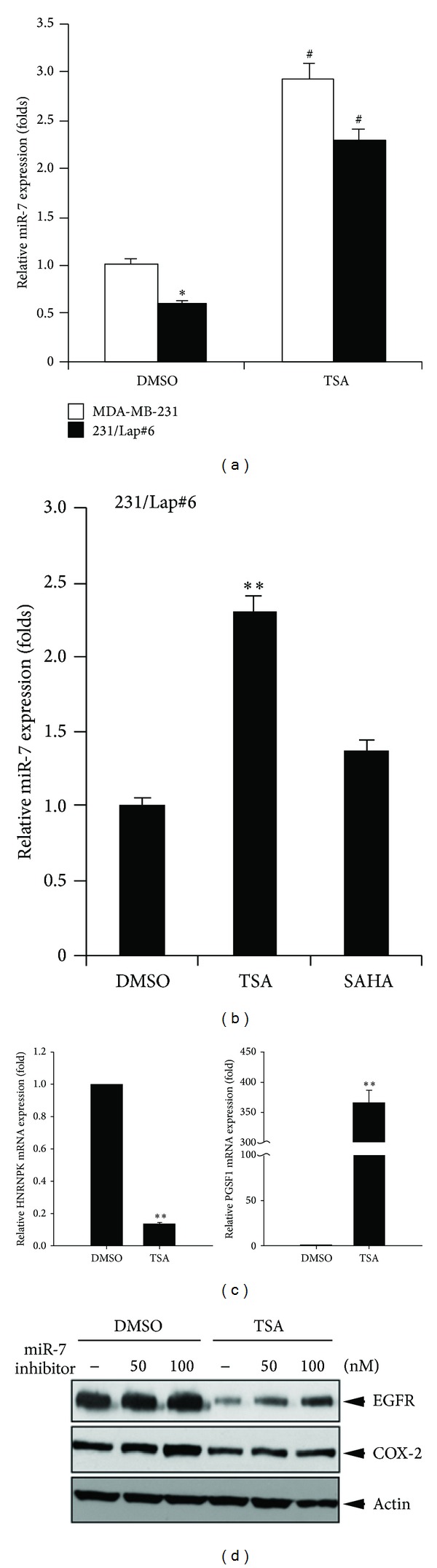
TSA induced miR-7 to suppress EGFR expression. (a)–(c) MDA-MB-231 and 231/Lap#6 cells were treated with 1 *μ*M TSA or SAHA for 24 hrs and then subjected to total RNA extraction. The levels of miR-7 and its host genes were measured by RT-qPCR analysis. (d) 231/Lap#6 cells were transfected with increasing doses of miR-7 inhibitor followed by treatment with TSA. Total lysates were prepared and subjected to Western blot.

MicroRNAs (miRNAs), a class of endogenous 17–24 base-long single-stranded, noncoding RNAs, widely regulate gene expression via targeting the 3′ untranslated region (UTR) in a sequence-specific manner. MicroRNA-7 (miR-7) has been reported to target the 3′UTR of *EGFR* mRNA and cause its degradation [36–38]. Our previous study also demonstrated that the elevation of EGFR expression is due to the downregulation of miR-7 in 231/Lap clones as compared with MDA-MB-231 cells. Thus, the possibility that TSA reduced EGFR expression in 231/Lap cells through induction of miR-7 was further addressed. To this end, the inhibitory effect of TSA on EGFR 3′UTR activity was examined in both parental and lapatinib-treated clones of MDA-MB-231 cells. In consistent with our previous results, the 3′UTR activity of EGFR was higher in 231/Lap#6 cells than in the parental cells (Figure 5(a)). Treatment with TSA 1 *μ*M for 24 hours can significantly suppress the EGFR 3′UTR activity in both cells (Figure 5(a)). However, the EGFR 3′UTR activity in 231/Lap#6 cells was not suppressed by 1 *μ*M SAHA (Figure 5(b)). Furthermore, the increase in miR-7 expression in both parental and lapatinib-treated MDA-MB-231 cells was also observed after treatment with TSA for 8 hours in quantitative RT-PCR assays (Figure 6(a)). However, the induction of miR-7 was not observed in SAHA-treated cells (Figure 6(b)). MicroRNA-7 is an intronic miRNA encoded in the host genes, including heterogeneous nuclear ribonucleoprotein K (HNRNPK) (for miR-7-1) and pituitary gland specific factor 1 (PGSF1) (for miR-7-3). Our data further showed that treatment with TSA dramatically induces the mRNA level of PGSF1 but not HNRNPK (Figure 6(c)), suggesting that TSA may induce miR-7-3 level through transcriptionally upregulating PGSF1 expression to target EGFR 3′UTR activity. Indeed, transfection of 231/Lap#6 cells with miR-7 inhibitor can dose-dependently reverse TSA-reduced EGFR expression (Figure 6(d)), demonstrating that TSA may induce miR-7 expression to target EGFR protein expression.

## 4. Discussion

In addition to the promising efficacy in HER2-positive breast cancer, use of lapatinib in HER2-negative diseases, especially in triple-negative cancers due to its frequent EGFR overexpression, is of interest and being tested currently [21, 22] but has been found to elicit diverse effects in different subgroups [8, 14, 39, 40]. Our previous findings indicated that treatment with lapatinib enhanced EGFR protein level through downregulation of miR-7 without affecting EGFR promoter activity in MDA-MB-231 cells. The lapatinib-induced EGFR subsequently maintained the NF-*κ*B-mediated COX-2 expression through HuR-dependent mRNA stabilization. These events rendered the triple-negative breast cancer cells showing more aggressive and higher metastasis rate to lymph node and lung [23]. These results provided a possible molecular mechanism explaining how addition of lapatinib to chemotherapy worsens the clinical outcome in breast cancer patients with triple-negative and HER2/PgR-negative tumors [14]. In this current study, we further explored that the pan-HDAC inhibitor TSA but not SAHA suppressed lapatinib-induced EGFR expression in an HDAC3/7-independent manner in 231/Lap cells.

HDACs are associated with the progression of cancer and have been demonstrated to mediate migration and invasion of cancer cells through different mechanisms [41–43]. Although the different roles of HDAC isoforms in the tumor development and progression of various cancer types have been characterized, the modulation of HDAC expression in response to anticancer treatments and their involvement in the metastatic relapse after the treatment is less understood. Our results showed that treatment with lapatinib increased HDAC3 and HDAC7 expressions accompanied with hypoacetylations of histone H3 K9 and histone H2B K5 (Figure 2). Suppression of these elevated HDACs by TSA and SAHA can reduce the motility of the lapatinib-treated cells (unpublished data). Despite their common effect on COX-2 suppression, TSA and SAHA were surprisingly found to have different activity in suppressing EGFR expression (Figure 1), suggesting a unique mechanism underlying the TSA-induced EGFR suppression. Although treatment with SAHA at 5–15 *μ*M has been reported to suppress EGFR expression in MDA-MB-231 cells [44], the concentration of SAHA for EGFR suppression used in their study is far higher than the IC50 of SAHA for HDAC inhibition (0.01-0.02 *μ*M) [33, 34]. Several lines of evidences provided from this study further support that TSA suppressed EGFR expression in an HDAC-independent manner in both parental and lapatinib-treated cells. (1) While treatments with TSA and SAHA at 1 *μ*M can completely abolish the total HDAC activity (Figure 1(a)), only TSA but not SAHA at the same concentration repressed EGFR expression in both parental and lapatinib-treated cells (Figures 1(b) and 1(c)). (2) Silence of HDAC3 or HDAC7 did not affect the protein (Figures 3(a) and 3(b)) and mRNA (Figure 3(c)) levels of EGFR in 231/Lap#6 cells. (3) Treatment of 231/Lap#6 cells with 1 *μ*M TSA suppressed the histone deacetylase activities of HDAC3 and HDAC7 (Figure 3(d)), but overexpression of myc-HDAC3 (Figure 3(e)) or myc-HDAC7 (Figure 3(f)) did not restore the EGFR expression in 231/Lap#6 cells. (4) HDAC6, a cytoplasmic class IIb deacetylase, was found to negatively regulate EGFR endocytosis and lysosomal degradation by controlling the acetylation status of *α*-tubulin [45]. When 231/Lap cells were treated with lysosomal and proteasomal inhibitors, the EGFR protein level was slightly enhanced, indicating that the lysosomal and proteasomal degradations indeed were involved in the turnover of EGFR. Although TSA induced tubulin acetylation, however, pretreatment with these lysosome or proteasome inhibitors did not prevent the TSA-reduced EGFR expression (Figure 4), ruling out the possibility that TSA decreases EGFR expression through targeting HDAC6-reduced EGFR endocytosis in 231/Lap#6 cells. These observations indicate that TSA may suppress EGFR independent of its HDAC inhibition activity.

In our previous study, treatment with lapatinib can downregulate miR-7 to target EGFR mRNA 3′UTR and thereby result in the derepression of EGFR expression in the 231/Lap cells. Our current data further revealed that treatment with TSA increased the expression of miR-7 in both parental and lapatinib-treated MDA-MB-231 cells. Transfection of miR-7 inhibitor dose-dependently prevented TSA-induced EGFR suppression (Figure 6(d)). However, treatment with SAHA or silence of HDAC3 and HDAC7 by siRNA did not affect the miR-7 expression (Figure 6(b) and data not shown), further suggesting that TSA may induce miR-7 expression through an HDAC-independent manner. Microarray results revealed that TSA altered expressions of many microRNAs involving tumor suppression, antimetastasis, and antiepithelial-mesenchymal transition (EMT) in apoptosis resistant MCF-7TN-R cells [46] or that involving general metabolisms in primary rat hepatocytes [47]. In contrast to inducing microRNA expression, TSA was also found to suppress miR-106b-93-25 cluster expression to inhibit proliferation and induce apoptosis in human endometrial cancer [48]. These results revealed that TSA may directly or indirectly regulate microRNA expressions through HDAC inhibition. However, the alteration of miR-7 by TSA was not found in these literatures, suggesting that cell contents may be critical for the regulation of microRNAs by TSA. In most cases, TSA and SAHA have similar effects on the regulation of gene and microRNA expressions [49]. Although both SAHA and TSA are derivatives of the hydroxamic acid and are structurally related to each other, acquisition of resistance to TSA or SAHA showed different dependence on MLH expression status [50]. Our data also showed that TSA but not SAHA suppressed EGFR expression through induction of miR-7. These observations suggest that actions of SAHA and TSA are different in some areas. Although our data suggest that miR-7 plays a critical role in TSA-mediated EGFR suppression, miR-7 inhibitor cannot totally restore EGFR expression in lapatinib-treated cells in response to TSA treatment, implying that, in addition to microRNA-7, other mechanisms underlying the TSA-mediated EGFR suppression cannot be ruled out. Treatment with HDAC inhibitor has been previously found to decrease EGFR mRNA and promoter activity by dissociation of transcription factor SP1 from the EGFR promoter around the transcription start site of EGFR gene in colorectal cancer cells [30]. Therefore, the Sp1 suppression and the HDAC-independent microRNA-7 induction may be both required for the TSA-mediated EGFR inhibition.

In conclusion, our data uncovered a unique activity of TSA in inducing miR-7 expression. In distinction to its structural relative SAHA, TSA suppressed EGFR 3′UTR activity to attenuate its protein expression independently of HDAC inhibition in lapatinib-treated breast cancer cells. These results suggest a possible off-target activity of TSA in suppressing EGFR expression.

## Abbreviations

TSA: Trichostatin A
SAHA: Suberoylanilide hydroxamic acid
EGFR: Epidermal growth factor receptor
RTKs: Receptor tyrosine kinases
miR/miRNA: MicroRNA
HDAC: Histone deacetylase.
